# Supplementary material for: Expansion of outer cortical CUX2 neurons requires adaptations for DNA repair
Source: Nature. 2026 Apr 1;653(8115):819–30. doi: 10.1038/s41586-026-10290-4 (PMC13190340; doi:10.1038/s41586-026-10290-4)

nature portfolio

https://doi.org/10.1038/s41586-026-10290-4

Supplementary information

Expansion of outer cortical CUX2 neurons requires adaptations for DNA repair

In the format provided by the authors and unedited

Supplementary Figure 1. Raw data and uncropped gels.

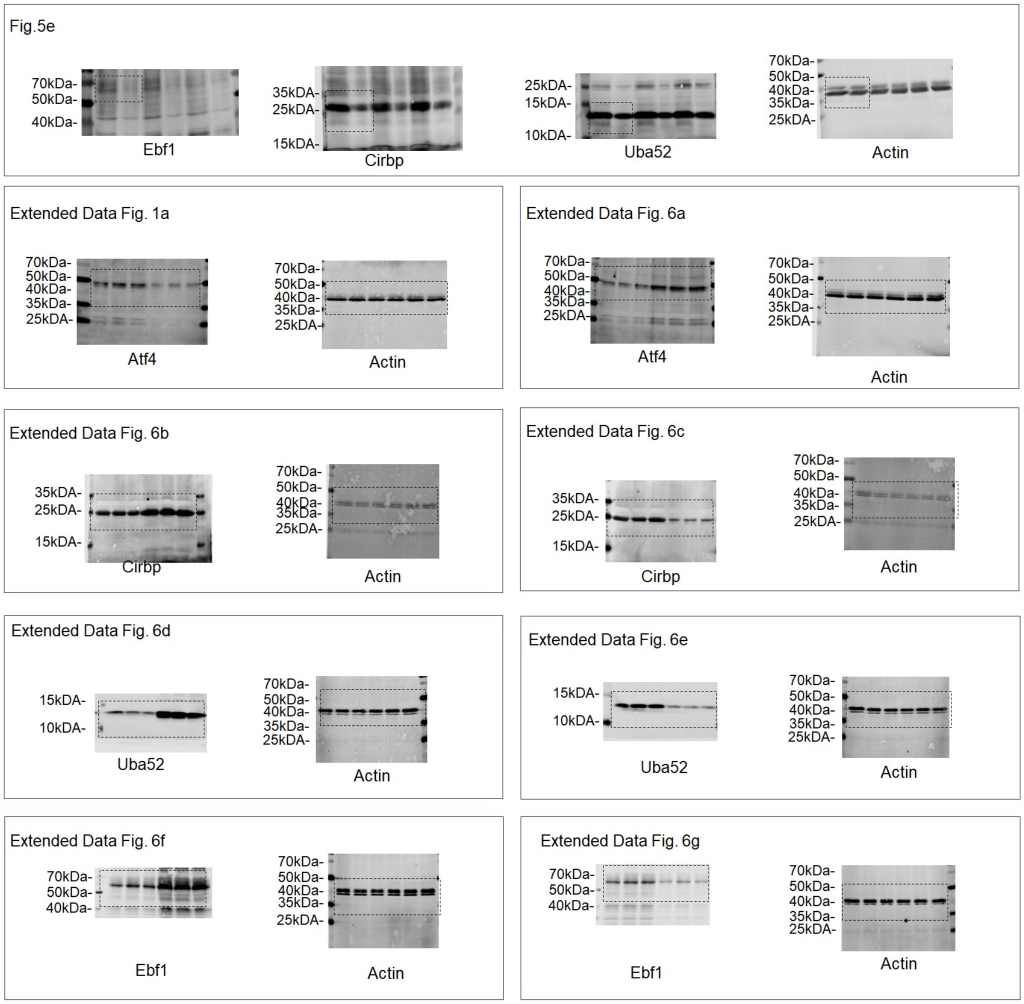

Supplement: Supplementary file 1 — Uncropped western blot images corresponding to Fig. 5e, Extended Data Fig. 1a and Extended Data Fig. 6. Data are derived from n = 3 independent experiments. Actin serves as a loading control. Black boxes indicate the lanes shown in the main and extended data figures [file 41586_2026_10290_MOESM1_ESM.pdf]
