# Supplementary material for: Expansion of outer cortical CUX2 neurons requires adaptations for DNA repair
Source: Nature. 2026 Apr 1;653(8115):819–30. doi: 10.1038/s41586-026-10290-4 (PMC13190340; doi:10.1038/s41586-026-10290-4)
Supplement: Supplementary file 2 — Reporting Summary [file 41586_2026_10290_MOESM2_ESM.pdf]

nature portfolio

Corresponding author(s): Stephen P.J. Fancy
David H. Rowitch

Last updated by author(s): Feb 5, 2026

Reporting Summary

Nature Portfolio wishes to improve the reproducibility of the work that we publish. This form provides structure for consistency and transparency in reporting. For further information on Nature Portfolio policies, see our Editorial Policies and the Editorial Policy Checklist.

Statistics

For all statistical analyses, confirm that the following items are present in the figure legend, table legend, main text, or Methods section.

n/a	Confirmed	
☐	☒	The exact sample size (*n*) for each experimental group/condition, given as a discrete number and unit of measurement
☐	☒	A statement on whether measurements were taken from distinct samples or whether the same sample was measured repeatedly
☐	☒	The statistical test(s) used AND whether they are one- or two-sided<br>*Only common tests should be described solely by name; describe more complex techniques in the Methods section.*
☒	☐	A description of all covariates tested
☐	☒	A description of any assumptions or corrections, such as tests of normality and adjustment for multiple comparisons
☐	☒	A full description of the statistical parameters including central tendency (e.g. means) or other basic estimates (e.g. regression coefficient) AND variation (e.g. standard deviation) or associated estimates of uncertainty (e.g. confidence intervals)
☐	☒	For null hypothesis testing, the test statistic (e.g. *F*, *t*, *r*) with confidence intervals, effect sizes, degrees of freedom and *P* value noted<br>*Give P values as exact values whenever suitable.*
☒	☐	For Bayesian analysis, information on the choice of priors and Markov chain Monte Carlo settings
☒	☐	For hierarchical and complex designs, identification of the appropriate level for tests and full reporting of outcomes
☒	☐	Estimates of effect sizes (e.g. Cohen's *d*, Pearson's *r*), indicating how they were calculated

Our web collection on statistics for biologists contains articles on many of the points above.

Software and code

Policy information about availability of computer code

Data collection	Imaging data was collected using Zeiss Zen software (Blue edition 2.6 pro)for Zeiss Apotome, Western Blot data was collected using LICOR odyssey system with the Image Studio software(ver 5.2), BioTek Synergy H4 with Gen5 Data Analysis Software (ver 3.11) was used to collect the data of luciferase reporter assay, qPCR data was collected with ABI QuantStudio 5 with QuantStudio Design& Analysis Software(v1.4.3), ISH and Nissl staining data were collected with ZEISS Axioscan and Zeiss Zen software(v3.7 slidescan)
Data analysis	All the data were analysed using Excel 2016 (ver 2511) GraphPad Prism 10.6.0, Images were analysed using Zeiss Zen (Blue edition 2.6 pro) or Image J(ver. 2.9.0) software. Single-nucleus RNA-seq data were processed using Cell Ranger (v7.0.1, 10x Genomics) for read alignment and gene counting against the mm10-2020-A reference genome. Ambient RNA contamination and barcode swapping were removed using CellBender (v0.3.0). Downstream analyses were performed primarily in Scanpy (v1.8.1), including highly variable gene selection, differential gene expression analysis, and gene set scoring. Cell clustering and batch correction were carried out using DESC (v2.1.1). Differential expression was assessed using the Wilcoxon rank-sum test implemented in Scanpy. Volcano plots were generated using ggplot2 in R (v3.4.4). Pathway and gene ontology enrichment analyses were performed using the clusterProfiler R package (v4.14.6). Protein–protein interaction analysis was conducted using STRING (v11.5) and then visualized by GOenrich package in clusterProfiler(version 4.14.6) with a network diagram.

For manuscripts utilizing custom algorithms or software that are central to the research but not yet described in published literature, software must be made available to editors and reviewers. We strongly encourage code deposition in a community repository (e.g. GitHub). See the Nature Portfolio guidelines for submitting code & software for further information.

Data

Policy information about availability of data

All manuscripts must include a data availability statement. This statement should provide the following information, where applicable:

- Accession codes, unique identifiers, or web links for publicly available datasets
- A description of any restrictions on data availability
- For clinical datasets or third party data, please ensure that the statement adheres to our policy

Single-nucleus RNA sequencing data from E11.5 mouse brains generated in this study have been deposited in the Gene Expression Omnibus (GEO) under accession number GSE314470. Source data are provided with this paper. All other data supporting the findings of this study are available from the corresponding author upon reasonable request.

Research involving human participants, their data, or biological material

Policy information about studies with human participants or human data. See also policy information about sex, gender (identity/presentation), and sexual orientation and race, ethnicity and racism.

Reporting on sex and gender	No
Reporting on race, ethnicity, or other socially relevant groupings	No
Population characteristics	Postmortem fetal tissue (GW15 and GW17) were used in this study for RNAscope. For GW15 sample, the age is 15 Gestational Weeks, PMI 15 hours, Clinical history: Cervical insufficiency, Neuropathological diagnosis: Control. For GW17 sample, the age is 17 Gestational Weeks, PMI 1hour 20mins, Clinical history: Hypoplastic left heart syndrome, Neuropathological diagnosis: Control. Listed as Case No.5 and Case No.6 from published paper(PMID: 35084970).
Recruitment	Human specimens were collected from autopsy, with previous patient consent to institutional ethical regulations of the University of California San Francisco Committee on Human Research as previously reported(PMID: 26798014, PMID: 35084970), and the sample been used in the published paper and listed as Case No.5 and Case No.6(PMID: 35084970).
Ethics oversight	Human specimens were collected from autopsy, with previous patient consent to institutional ethical regulations of the University of California San Francisco Committee on Human Research as previously reported(PMID: 26798014, PMID: 35084970)

Note that full information on the approval of the study protocol must also be provided in the manuscript.

Field-specific reporting

Please select the one below that is the best fit for your research. If you are not sure, read the appropriate sections before making your selection.

☒ Life sciences ☐ Behavioural & social sciences ☐ Ecological, evolutionary & environmental sciences

For a reference copy of the document with all sections, see nature.com/documents/nr-reporting-summary-flat.pdf

Life sciences study design

All studies must disclose on these points even when the disclosure is negative.

Sample size	Minimum sample sizes were estimated based on previously published studies(PMID: 30787442, PMID: 41339559), and our experiences(PMID: 26798014, PMID: 36384142). Normally, at least 3 samples per group were used for statistical analyses.
Data exclusions	No exclusions
Replication	For all the graphical representations of the data, the number of and types of replicates used are mentioned in their respective figure legends and methods. All the experiments were performed with 3 or more biological replicates.
Randomization	Mice were assigned randomly to the experimental groups. For non-mouse experiments, samples were assigned to different experimental groups according to pre-defined experimental conditions. All samples were processed in parallel under identical conditions. To minimize potential batch effects, the order of sample assignment and data acquisition was balanced across groups.
Blinding	Blinding was employed for all analysis. During the data collection process, researchers were unaware of the group assignments. Samples were independently coded, and data collection was performed without knowledge of the genotype or experimental conditions. Group assignment information was only revealed after data collection and initial quantitative analysis were completed.

Reporting for specific materials, systems and methods

We require information from authors about some types of materials, experimental systems and methods used in many studies. Here, indicate whether each material, system or method listed is relevant to your study. If you are not sure if a list item applies to your research, read the appropriate section before selecting a response.

Materials & experimental systems

n/a	Involved in the study	
☐	☒	Antibodies
☐	☒	Eukaryotic cell lines
☒	☐	Palaeontology and archaeology
☐	☒	Animals and other organisms
☒	☐	Clinical data
☒	☐	Dual use research of concern
☒	☐	Plants

Methods

n/a	Involved in the study	
☒	☐	ChIP-seq
☒	☐	Flow cytometry
☒	☐	MRI-based neuroimaging

Antibodies

Antibodies used

For the immunostaining: GFP was detected with Anti-Green Fluorescent Protein (GFP) Antibody GFP-1020 (Aves) at 1:1000 dilution, mouse Pax6 was detected with Anti-PAX6 Antibody AB2237 (Millipore) at 1:500 dilution, mouse Tbr2 was detected with Anti-TBR2 / Eomes antibody ab23345 (Abcam) at 1:500 dilution, mouse Tbr1 was detected with Anti-TBR1 antibody ab31940 (Abcam) at 1:1000 dilution, mouse Tuj1 was detected with Anti-β-Tubulin III Antibody T2200 (Sigma) at 1:1000 dilution, mouse Satb2 was detected with Anti-SATB1 + SATB2 antibody [SATBA4B10] - C-terminal ab51502 (Abcam) at 1:500 dilution, mouse Ctip2 was detected with Anti-Ctip2 antibody [25B6] ab18465 (Abcam) at 1:1000 dilution, mouse Cux1+Cux2 was detected with Anti-CUX1+CUX2 antibody [EPR26509-154] ab309139 (Abcam) at 1:500 dilution, mouse Calretinin was detected with Anti-Calretinin Antibody, clone 6B8.2 MAB1568 (Millipore) at 1:500 dilution, mouse Parvalbumin was detected with Anti-Parvalbumin Antibody MAB1572 (Millipore) at 1:500 dilution, mouse Calbindin was detected with Anti- Calbindin antibody CB38a (Swant) at 1:500 dilution, human PAX6 was detected with anti-Pax-6 Antibody 901301 (Biolegend) at 1:500 dilution, mouse gamma H2A.X was detected with Anti-gamma H2A.X (phospho S139) antibody ab2893 (abcam) at 1:500 dilution or Anti-phospho-Histone H2A.X (Ser139) Antibody, clone JBW301 05-636 (Millipore) at 1:500 dilution, mouse Cleaved Caspase-3 was detected with Anti-Cleaved Caspase-3 (Asp175) Antibody 9661(cell signaling technology) at 1:400 dilution, mouse Phospho-KAP-1 (Ser824) was detected with Anti-Phospho-KAP-1 (Ser824) Polyclonal antibody A300-767A (Bethyl Laboratories) at 1:1000 dilution, mouse P53 was detected with Anti-p53 (1C12) Mouse Monoclonal Antibody 2524 (cell signaling technology) at 1:500 dilution, mouse PCNA was detected with Anti-PCNA (PC10) Mouse Monoclonal Antibody 2586 (cell signaling technology) at 1:500 dilution, mouse Ki67 was detected with antibody 550609 (BD Biosciences) at 1:500 dilution, mouse 53bp1 was detected with Anti-53BP1 Antibody NB100-304 (Novus Biologicals) at 1:500 dilution, mouse DNA-RNA Hybrid S9.6 was detected with Anti-DNA-RNA Hybrid [S9.6] Antibody ENH001 (Kerafast) at 1:500 dilution, mouse p-ATM(Ser1981) was detected with Anti-phospho-ATM (Ser1981) Antibody, clone 10H11.E12 05-740 (Millipore Sigma) at 1:500 dilution. Mouse phh3 was detected with Phospho-Histone H3 (Ser10) Antibody 9701 (cell signaling technology) at 1:500 dilution. Mouse Nestin was detected with Anti-Nestin Antibody, clone rat-401 MAB353 (Millipore Sigma) at 1:500 dilution. Mouse Sox2 was detected with Anti-SOX2 antibody [EPR3131] ab92494 (abcam) at 1:500 dilution.
For immunoblotting: Beta-Actin was detected with Anti-Beta Actin Monoclonal antibody 66009-1-Ig (proteintech) at 1:1000 dilution, Ebf1 was detected with Anti-EBF-1 Antibody AB10523 (Millipore) at 1:500 dilution, UBA52 was detected with Anti-UBA52 Polyclonal antibody 18039-1-AP (proteintech) at 1:500 dilution, Cirbp was detected with CIRBP Polyclonal antibody 10209-2-AP (proteintech) at 1:500 dilution, Atf4 was detected with Anti-ATF-4 (D4B8) Rabbit Monoclonal Antibody 11815 (cell signaling technology) at 1:500 dilution.

For ChIP-qPCR: Anti-ATF-4 (D4B8) Rabbit Monoclonal Antibody 11815 (cell signaling technology) used at 1:50 dilution.

For the ISH experiment, Anti-Digoxigenin-AP, Fab fragments been used at 1;1500 dilution, 11093274910(ROCHE)

Secondary antibodies:
Goat anti-Chicken IgY (H+L) Secondary Antibody, Alexa Fluor™ 488 ; A11039; 1:1000 dilution; Invitrogen
Goat anti-Mouse IgG (H+L) Cross-Adsorbed Secondary Antibody, Alexa Fluor 488; A11001; 1:1000 dilution; Invitrogen
Goat anti-Mouse IgG (H+L) Cross-Adsorbed Secondary Antibody, Alexa Fluor 594; A11005; 1:1000 dilution; Invitrogen
Goat anti-Mouse IgG (H+L) Secondary Antibody, Alexa Fluor® 647 conjugate; A21236; 1:1000 dilution; Invitrogen
Goat anti-Rabbit IgG (H+L) Cross-Adsorbed Secondary Antibody, Alexa Fluor 488; A11008; 1:1000 dilution; Invitrogen
Goat anti-Rabbit IgG (H+L) Cross-Adsorbed Secondary Antibody, Alexa Fluor 594; A11012; 1:1000 dilution; Invitrogen
Goat anti-Rabbit IgG (H+L) Highly Cross-Adsorbed Secondary Antibody, Alexa Fluor 647; A21245; 1:1000 dilution; Invitrogen
Goat anti-Rat IgG (H+L) Cross-Adsorbed Secondary Antibody, Alexa Fluor™ 488, Invitrogen™; A11006; 1:1000 dilution; Invitrogen
Goat anti-Rat IgG (H+L) Cross-Adsorbed Secondary Antibody, Alexa Fluor 594; A11007; 1:1000 dilution; Invitrogen
Goat anti-Rat IgG (H+L) Secondary Antibody, Alexa Fluor® 647 conjugate; A21247; 1:1000 dilution; Invitrogen
Highly Cross-Adsorbed Goat (Polyclonal) Anti-Mouse IgG (H+L) Antibody Conjugated to IRDye 680RD; 926-68070; 1;20000 dilution; LICORbio;
Highly cross-adsorbed goat (polyclonal) anti-rabbit IgG (H+L) antibody conjugated to IRDye 680RD; 926-68071; ;20000 dilution; LICORbio;

Validation

All of the antibodies used were chosen from published research and have been validated by the manufacturer for the specific species and application. All information is listed here:
1: Anti-Green Fluorescent Protein (GFP) GFP-1020 (Aves): https://www.antibodiesinc.com/products/anti-green-fluorescent-protein-antibody-gfp?utm_source=citeab&utm_medium=affiliate&utm_campaign=product
2: Anti-PAX6 Antibody AB2237 (Millipore) https://www.sigmaaldrich.com/US/en/product/mm/ab2237
3: Anti-TBR2 / Eomes antibody ab23345 (Abcam)
https://www.abcam.com/en-us/products/primary-antibodies/tbr2-eomes-antibody-ab23345
4: Anti-TBR1 antibody ab31940 (Abcam)
https://www.abcam.com/en-us/products/primary-antibodies/tbr1-antibody-ab31940
5: Anti-β-Tubulin III Antibody T2200 (Sigma)
https://www.sigmaaldrich.com/US/en/product/sigma/t2200?srsltid=AfmBOopCTJbYTjkeTDPBEMiUK0Cz3DnWg1J7rHukdfANqxpWUvW9QC0f
6: Anti-SATB1 + SATB2 antibody [SATBA4B10] - C-terminal ab51502 (Abcam)
https://www.abcam.com/en-us/products/primary-antibodies/satb1-satb2-antibody-satba4b10-c-terminal-ab51502
7: Anti-Ctip2 antibody [25B6] ab18465 (Abcam)
https://www.abcam.com/en-us/products/primary-antibodies/ctip2-antibody-25b6-ab18465
8: Anti-CUX1+CUX2 antibody [EPR26509-154] ab309139 (Abcam)
https://www.abcam.com/en-us/products/primary-antibodies/cux1cux2-antibody-epr26509-154-ab309139
9: Anti-Calretinin Antibody, clone 6B8.2 MAB1568 (Millipore)
https://www.sigmaaldrich.com/US/en/product/mm/mab1568?srsltid=AfmBOoourzDuL7C-QliiWfRK2-JH0fQooDbIJYninIQpWAM0IlQXDSrf
10: Anti-Parvalbumin Antibody MAB1572 (Millipore)
https://www.sigmaaldrich.com/US/en/product/mm/mab1572?srsltid=AfmBOooU7Sxl7jSfVjjXQFlheVcVkUsgYsyeXsPk0xiQDntEyjKsz3xB
11: Anti- Calbindin antibody CB38a (Swant)
https://shop.swant.com/cb38a-calbindin.html
12: anti-Pax-6 Antibody 901301 (Biolegend)
https://www.biolegend.com/nl-nl/products/purified-anti-pax-6-antibody-11511?displayInline=true&filename=Purified%20anti-Pax-6%20Antibody.pdf&leftRightMargin=15&pdf=true&topBottomMargin=15&v=20250227010954
13: Anti-gamma H2A.X (phospho S139) antibody ab2893 (abcam)
https://www.abcam.com/en-us/products/primary-antibodies/gamma-h2ax-phospho-s139-antibody-ab2893
14: Anti-phospho-Histone H2A.X (Ser139) Antibody, clone JBW301 05-636 (Millipore)
https://www.sigmaaldrich.com/US/en/product/mm/05636?srsltid=AfmBOor46JY6MrIeXmATEXcY7DRo2Pq2QBdlMlRh7OE-UxNWKL7g7esd
15: Anti-Cleaved Caspase-3 (Asp175) Antibody 9661(cell signaling technology)
https://www.cellsignal.com/products/primary-antibodies/cleaved-caspase-3-asp175-antibody/9661?srsltid=AfmBOorED8btjid1f1KdGY2raxGyMt4VrQqxOkhxT0sl9VmpAit1staw
16: Anti-Phospho-KAP-1 (Ser824) Polyclonal antibody A300-767A (Bethyl Laboratories)
https://www.thermofisher.com/antibody/product/Phospho-KAP-1-Ser824-Antibody-Polyclonal/A300-767A
17: Anti-p53 (1C12) Mouse Monoclonal Antibody 2524 (cell signaling technology)
https://www.cellsignal.com/products/primary-antibodies/p53-1c12-mouse-monoclonal-antibody/2524?srsltid=AfmBOoqgnOE0bWhEDqWv1v73fciS9mN6Iiq7aK-AOpDx2XrcYRQjZHpj
18: Anti-PCNA (PC10) Mouse Monoclonal Antibody 2586 (cell signaling technology)
https://www.cellsignal.com/products/primary-antibodies/pcna-pc10-mouse-monoclonal-antibody/2586?srsltid=AfmBOooJR-o0wKAsUyA9k5JYHNoOB4XAdFiW1HHGoZO61XaVRugrTKb0
19: Anti-53BP1 Antibody NB100-304 (Novus Biologicals)
https://www.novusbio.com/products/53bp1-antibody_nb100-304?srsltid=AfmBOoqXirQRpT3GLjGQZ7PD_PNLhPZwRUC4r-jWVaKtFn9R3H1pnceA
20: Anti-DNA-RNA Hybrid [S9.6] Antibody ENH001 (Kerafast)
https://www.kerafast.com/productgroup/432/anti-dna-rna-hybrid-s96-antibody
21: Anti-phospho-ATM (Ser1981) Antibody, clone 10H11.E12 05-740 (Millipore Sigma)
https://www.sigmaaldrich.com/US/en/product/mm/05740?srsltid=AfmBOoqwv_9ymkVKzucidMuPt1UgRxMOZkXurWdxhwedwV6CY7uu8Xh8
22: Phospho-Histone H3 (Ser10) Antibody 9701 (cell signaling technology)
https://www.cellsignal.com/products/primary-antibodies/phospho-histone-h3-ser10-antibody/9701?srsltid=AfmBOor-gfGf97hsjIB4UH9EJ8aH2zrX4RSr1ZJx68YW94JhkBRSui5n
23: Anti-Nestin Antibody, clone rat-401 MAB353 (Millipore Sigma)
https://www.sigmaaldrich.com/US/en/product/mm/mab353?srsltid=AfmBOopoxvMbH0d7rpdoxh9-gYXjGkttSJ8h18j5Yq8RxRQWJ44AKqW-
24: Anti-SOX2 antibody [EPR3131] ab92494 (abcam)
https://www.abcam.com/en-us/products/primary-antibodies/sox2-antibody-epr3131-ab92494
25: Anti-Beta Actin Monoclonal antibody 66009-1-Ig (proteintech)
https://www.ptglab.com/products/Pan-Actin-Antibody-66009-1-Ig.htm?srsltid=AfmBOorrHJR5l4OquiWo9mFX7RN3L4BFWQfzDs3VdfROVqN_H21vZYvl
26: Anti-EBF-1 Antibody AB10523 (Millipore)

https://www.sigmaaldrich.com/US/en/product/mm/ab10523?srsltid=AfmBOorckej_8O8PUbDeZ6iGNipTPzdi0yxmetE88QFMk_90aWjNrUG4
27: Anti-UBA52 Polyclonal antibody 18039-1-AP (proteintech)
https://www.ptglab.com/products/UBA52-Antibody-18039-1-AP.htm?srsltid=AfmBOoqKzpFGxM2V8uvh_sMW0vo4G4YsV2oS3JPt7MRQTFZCnGGOGTOE
28: CIRBP Polyclonal antibody 10209-2-AP (proteintech)
https://www.ptglab.com/products/CIRBP-Antibody-10209-2-AP.htm?srsltid=AfmBOookLo0lo4_mNkeGy9sgVA3nPNoVt8fpWTJ_LkWJjzwJu0ITKRp1
29: Anti-ATF-4 (D4B8) Rabbit Monoclonal Antibody 11815 (cell signaling technology)
https://www.cellsignal.com/products/primary-antibodies/atf-4-d4b8-rabbit-monoclonal-antibody/11815?srsltid=AfmBOop2flu7FRJfrn5CJwrmp5LgefIJBzzy8GfvpundmXHyebMoJ5yE
30: Anti-Digoxigenin-AP, Fab fragments, 11093274910(ROCHE)
https://www.sigmaaldrich.com/US/en/product/roche/11093274910?srsltid=AfmBOoql-NORqVQ2jaa9uuU9GMv55yWRynlBx5-Jrfae240YHtWd-jgf
31: Goat anti-Chicken IgY (H+L) Secondary Antibody, Alexa Fluor™ 488 ; A11039
https://www.thermofisher.com/antibody/product/A11039.html?ef_id=Cj0KCQiAkPzLBhD4ARIsAGfah8hiVZeF-LweJHmhyhiF9AMb_adQY-kAtLIIM7zVwEgEL6TTyCbmteoaAt1xEALw_wcB:G:s&s_kwcid=AL!3652!3!!!!x!!&gad_source=1&gad_campaignid=23463429566&gbraid=0AAAAADxi_GRlQD99T3m6b32wsvWiftVl1&gclid=Cj0KCQiAkPzLBhD4ARIsAGfah8hiVZeF-LweJHmhyhiF9AMb_adQY-kAtLIIM7zVwEgEL6TTyCbmteoaAt1xEALw_wcB
31: Goat anti-Mouse IgG (H+L) Cross-Adsorbed Secondary Antibody, Alexa Fluor 488; A11001
https://www.thermofisher.com/antibody/product/Goat-anti-Mouse-IgG-H-L-Cross-Adsorbed-Secondary-Antibody-Polyclonal/A-11001
32: Goat anti-Mouse IgG (H+L) Cross-Adsorbed Secondary Antibody, Alexa Fluor 594; A11005
https://www.thermofisher.com/antibody/product/Goat-anti-Mouse-IgG-H-L-Cross-Adsorbed-Secondary-Antibody-Polyclonal/A-11005
33: Goat anti-Mouse IgG (H+L) Secondary Antibody, Alexa Fluor® 647 conjugate; A21236
https://www.thermofisher.com/antibody/product/Goat-anti-Mouse-IgG-H-L-Highly-Cross-Adsorbed-Secondary-Antibody-Polyclonal/A-21236
34: Goat anti-Rabbit IgG (H+L) Cross-Adsorbed Secondary Antibody, Alexa Fluor 488; A11008
https://www.thermofisher.com/antibody/product/Goat-anti-Rabbit-IgG-H-L-Cross-Adsorbed-Secondary-Antibody-Polyclonal/A-11008
35: Goat anti-Rabbit IgG (H+L) Cross-Adsorbed Secondary Antibody, Alexa Fluor 594; A11012
https://www.thermofisher.com/antibody/product/Goat-anti-Rabbit-IgG-H-L-Cross-Adsorbed-Secondary-Antibody-Polyclonal/A-11012
36: Goat anti-Rabbit IgG (H+L) Highly Cross-Adsorbed Secondary Antibody, Alexa Fluor 647; A21245
https://www.thermofisher.com/antibody/product/Goat-anti-Rabbit-IgG-H-L-Highly-Cross-Adsorbed-Secondary-Antibody-Polyclonal/A-21245
37: Goat anti-Rat IgG (H+L) Cross-Adsorbed Secondary Antibody, Alexa Fluor™ 488, Invitrogen™; A11006
https://www.thermofisher.com/antibody/product/Goat-anti-Rat-IgG-H-L-Cross-Adsorbed-Secondary-Antibody-Polyclonal/A-11006
38: Goat anti-Rat IgG (H+L) Cross-Adsorbed Secondary Antibody, Alexa Fluor 594; A11007
https://www.thermofisher.com/antibody/product/Goat-anti-Rat-IgG-H-L-Cross-Adsorbed-Secondary-Antibody-Polyclonal/A-11007
39: Goat anti-Rat IgG (H+L) Secondary Antibody, Alexa Fluor® 647 conjugate; A21247
https://www.thermofisher.com/antibody/product/Goat-anti-Rat-IgG-H-L-Cross-Adsorbed-Secondary-Antibody-Polyclonal/A-21247
40: Highly Cross-Adsorbed Goat (Polyclonal) Anti-Mouse IgG (H+L) Antibody Conjugated to IRDye 680RD; 926-68070; 1;20000 dilution; LICORbio;
https://www.licorbio.com/support/contents/reagents/irdye-secondary-antibodies/680rd/goat-anti-mouse-igg.html
41: Highly cross-adsorbed goat (polyclonal) anti-rabbit IgG (H+L) antibody conjugated to IRDye 680RD; 926-68071
https://shop.licorbio.com/irdye-secondary-antibodies/irdye-680rd-goat-anti-rabbit-igg-secondary-antibody/

Eukaryotic cell lines

Policy information about cell lines and Sex and Gender in Research

Cell line source(s)	HEK293T (ATCC #CRL-3216) cell line was used for lenti-virus packaging. The 293T cell line, originally referred as 293tsA1609neo, is a highly transfectable derivative of human embryonic kidney 293 cells, and contains the SV40 T-antigen.
Authentication	HEK293T (ATCC #CRL-3216) cell line identity was authenticated by short tandem repeat (STR) profiling prior to use. STR profiling by ATCC(https://www.atcc.org/products/crl-3216).
Mycoplasma contamination	Lines tested negative for mycoplasma contamination
Commonly misidentified lines (See ICLAC register)	No.

Animals and other research organisms

Policy information about studies involving animals; ARRIVE guidelines recommended for reporting animal research, and Sex and Gender in Research

Laboratory animals	This study used mice at various developmental stages and includes both males and females.<br><br>The mice age in between 2 to 6 months old were used for the breeding.

All mice were handled in accordance with NIH guidelines and protocols approved by the UCSF Institutional Animal Care and Use Committee. Mice were housed under specific pathogen-free conditions in individually ventilated cages within a barrier facility on a 12-h light/dark cycle, with controlled temperature (68–79 °F) and humidity (30–70%). Housing density did not exceed five adult mice per cage; breeding cages (one male, up to two females) were maintained in a dedicated high-barrier area. Cages were changed weekly under laminar flow hoods, access was restricted with required PPE, and colony health was monitored using sentinel mice. Both sexes were used, no sex-specific differences were observed, and mice were randomly assigned to experimental groups.

C57BL/6 wild-type (WT) mice were obtained from The Jackson Laboratory (JAX:000664).

The Emx1-Cre line (B6.129S2Emx1tm1(cre)Krj/J, JAX:005628) has been previously described. These mice were crossed with Atf4 floxed mice to delete Atf4 specifically in the early embryonic cortex. To assess phenotypes after blocking cell death, Emx1-Cre mice were also crossed with Atf4 floxed and p53 null animals. In addition, Emx1-Cre mice were crossed with LSL-H2B-GFP mice for lineage tracing of Emx1+ cortical cells across developmental stages.

The Atf4fl/fl line (C57BL/6-Atf4tm1.1Cmad/J, JAX:033380) carries loxP sites flanking exons 2–3, which include the ATG start codon of the Atf4 gene. These mice have been previously described and were crossed with Emx1-Cre and/or p53 null mice to knockout the Atf4 expression.

The p53 null (p53-/-) line (B6.129S2-Trp53tm1Tyj/J, JAX:002101) carries a neomycin cassette replacing exons 2–6 (including the start codon) of the Trp53 gene. These mice were crossed with Emx1-Cre and Atf4 floxed mice to block p53-dependent cell death.
The LSL-H2B-GFP line (B6.Cg-Gt(ROSA)26Sortm8(CAG-HIST1H2BB/EGFP)Zjh/J JAX:036761) harbors a targeted mutation in the Gt(ROSA)26Sor locus with a loxP-flanked STOP cassette preventing transcription of a CAG promoter–driven enhanced green fluorescent protein (EGFP).

IUE experiment was done with time-plugged pregnant animal with ECM BTX830, according to previous published paper(PMID: 28524856).

Embryonic brains were collected from timed-pregnant mice at the indicated stages, and genotyping was performed using tissue from the same embryos.
For neonatal samples, the day of birth was designated postnatal day 0 (P0), and brains were collected at the indicated postnatal stages with genotyping performed on tissue from the same animals.

Wild animals	NO
Reporting on sex	Animals were used unbaised to sex.
Field-collected samples	No
Ethics oversight	All animal protocols were in accordance with the regulations of the National Institute of Health and approved by the University of California San Francisco Institutional Animal Care and Use Committee (IACUC).

Note that full information on the approval of the study protocol must also be provided in the manuscript.

Plants

Seed stocks	No
Novel plant genotypes	No
Authentication	No
