# Supplementary material for: Expansion of outer cortical CUX2 neurons requires adaptations for DNA repair
Source: Nature. 2026 Apr 1;653(8115):819–30. doi: 10.1038/s41586-026-10290-4 (PMC13190340; doi:10.1038/s41586-026-10290-4)
Supplement: Supplementary file 3 — Supplementary Table 1. List of DDR genes. DNA damage repair gene set. A comprehensive list of 698 DNA damage repair genes was assembled by merging genes annotated under GO:0006281 (DNA repair) and HALLMARK_DNA_REPAIR (M5898), and integrating this with the curated “Human DNA Repair Genes” list by R. Wood and M. Lowery. Supplementary Table 2. List of unfolded protein response (UPR) genes. UPR gene set. This set includes 116 genes associated with GO:0006986, sourced from the Mouse Genome Informatics (MGI) database. Supplementary Table 3. List of integrated stress response (ISR) genes. ISR gene set. Comprising 23 genes, this set is based on GO:0140467 from the MGI database. Supplementary Table 4. List of amino acid transport (AAT) genes. AAT gene set. This set includes 193 genes annotated under GO:0006865, sourced from the MGI database. Supplementary Table 5. List of peroxidase activity (PACT) genes. PACT gene set. Consisting of 64 genes, this set is derived from GO:0004601 (peroxidase activity), also sourced from the MGI database. Supplementary Table 6. List of NRF2 target genes. NRF2 target gene set. This set includes 37 genes identified as NRF2 targets, based on a curated list from a published review article (ref. 56). Supplementary Table 7. List of eukaryotic translation factor (ETF) genes. ETF gene set. This set includes 69 genes including initiation, elongation, termination and ribosome recycling factors. Supplementary Table 8. Used oligonucleotides. All oligonucleotide sequences used in this study, including primers for qPCR and ChIP–qPCR, shRNA sequences and primers used for cloning. [file 41586_2026_10290_MOESM3_ESM.pdf]

Supplementary Table 1: List of DNA damage response (DDR) genes

AAAS	BABAM1	CHAF1A	DYRK1B	FANCG	HERC2	KMT5C	MSH5	PARP10
ABL1	BABAM2	CHAF1B	EDF1	FANCI	HINFP	LIG1	MSH6	PARP2
ABRAXAS1	BACH1	CHCHD4	EEPD1	FANCL	HLTF	LIG3	MTA1	PARP3
ACTB	BARD1	CHD1L	EGFR	FANCM	HMCES	LIG4	MUS81	PARP9
ACTL6A	BCAM	CHD4	EID3	FBH1	HMGA1	MAD2L2	MUTYH	PARPBP
ACTL6B	BCAP31	CHEK1	EIF1B	FBXO6	HMGA2	MAJIN	NABP1	PAXIP1
ACTR2	BCCIP	CHEK2	ELL	FBXW7	HMGB1	MARF1	NABP2	PAXX
ACTR5	BCL7A	CHRNA4	EME1	FEN1	HMGN1	MBD4	NBN	PBRM1
ACTR8	BCL7B	CINP	EME2	FGF10	HPF1	MBTD1	NCBP2	PCLAF
ADA	BCL7C	CLP1	EMSY	FIGNL1	HPRT1	MC1R	NEIL1	PCNA
ADCY6	BLM	CLSPN	ENDOV	FLNB	HROB	MCM2	NEIL2	PDE4B
ADPRS	BOD1L1	CMPK2	ENY2	FMN2	HSF1	MCM3	NEIL3	PDE6G
ADRM1	BOLA2	COMMD1	EP400	FMR1	HSF2BP	MCM4	NFRKB	PDS5A
AK1	BRCA1	COX17	EPC1	FOXM1	HSPA1A	MCM5	NFX1	PDS5B
AK3	BRCA2	CSTF3	EPC2	FTO	HSPA1B	MCM6	NHEJ1	PHF10
ALKBH1	BRCC3	CUL4A	ERCC1	FUS	HUS1	MCM7	NIPBL	PIAS4
ALKBH2	BRD7	CUL4B	ERCC2	FZR1	HUS1B	MCM8	NME1	PIF1
ALKBH3	BRD8	CYREN	ERCC3	GCNA	HUWE1	MCM9	NME3	PML
ANKLE1	BRF2	DAD1	ERCC4	GEN1	IER3	MCMDC2	NME4	PMS1
AP5S1	BRIP1	DCLRE1A	ERCC5	GGN	IFFO1	MCRS1	NONO	PMS2
AP5Z1	BRME1	DCLRE1B	ERCC6	GINS2	IMPDH2	MDC1	NOP53	PNKP
APBB1	C14ORF39	DCLRE1C	ERCC6L2	GINS4	ING3	MEAF6	NPAS2	PNP
APEX1	CANT1	DCTN4	ERCC8	GMPR2	INIP	MEIOB	NPM1	POGZ
APEX2	CBX8	DDB1	ESCO2	GPX4	INO80	MEIOC	NPR2	POLA1
APLF	CCDC117	DDB2	ETAA1	GTF2A2	INO80B	MGME1	NSD2	POLA2
APRT	CCNO	DDX1	EXD2	GTF2B	INO80C	MGMT	NSMCE1	POLB
APTX	CDA	DDX11	EXO1	GTF2F1	INO80D	MLH1	NSMCE2	POLD1
ARID1A	CDC14B	DDX3X	EXO5	GTF2H1	INO80E	MLH3	NSMCE3	POLD2
ARID1B	CDC45	DEK	EYA1	GTF2H2	INTS3	MMS19	NSMCE4A	POLD3
ARID2	CDC5L	DGCR8	EYA2	GTF2H2C	ITPA	MMS22L	NT5C	POLD4
ARL6IP1	CDC7	DGUOK	EYA3	GTF2H3	JMY	MNAT1	NTHL1	POLDIP2
ASCC1	CDCA5	DHX9	EYA4	GTF2H4	KASH5	MORC2	NUCKS1	POLE
ASCC2	CDK2	DMAP1	FAAP100	GTF2H5	KAT2A	MORF4L1	NUDT16L1	POLE2
ASCC3	CDK7	DMC1	FAAP20	GTF3C5	KAT2B	MORF4L2	NUDT21	POLE4
ASF1A	CDK9	DNA2	FAAP24	GUK1	KAT5	MPG	NUDT9	POLG2
ATM	CDKN2D	DNTT	FAM111A	H2AC25	KAT7	MPND	OGG1	POLH
ATR	CEBPG	DOT1L	FAM168A	H2AX	KDM1A	MRE11	OOEP	POLI
ATRIP	CENPS	DPF1	FAN1	HCLS1	KDM2A	MRGBP	OTUB1	POLK
ATRX	CENPX	DPF2	FANCA	HDAC10	KDM4D	MRNIP	OTUB2	POLL
ATXN7	CEP164	DPF3	FANCB	HDAC9	KIF22	MRPL40	PALB2	POLM
ATXN7L3	CETN1	DTL	FANCC	HDGFL2	KIN	MSH2	PARG	POLN
AUNIP	CETN2	DTX3L	FANCD2	HELB	KLHL15	MSH3	PARK7	POLQ
AXIN2	CGAS	DUT	FANCF	HELQ	KMT5B	MSH4	PARP1	POLR1C

Supplementary Table 1 (continued): List of DNA damage response (DDR) genes

POLR1D	RAD54B	RUVBL1	SMC3	TAF6	UBE2A	XPA	CLK2
POLR2A	RAD54L	RUVBL2	SMC4	TAF6L	UBE2B	XPC	RDM1
POLR2C	RAD9A	SAC3D1	SMC5	TAF7	UBE2D3	XRCC1	PER1
POLR2D	RAD9B	SAMHD1	SMC6	TAF9	UBE2N	XRCC2	RPA4
POLR2E	RADX	SDCBP	SMCHD1	TAOK1	UBE2T	XRCC3	POLE3
POLR2F	RAE1	SEC61A1	SMG1	TAOK3	UBE2V1	XRCC4	PMS2P3
POLR2G	RALA	SEM1	SMUG1	TARBP2	UBE2V2	XRCC5	MPLKIP
POLR2H	RBBP8	SETD2	SNAPC4	TDG	UBE2W	XRCC6	NUDT18
POLR2I	RBX1	SETMAR	SNAPC5	TDP1	UBQLN4	XRN2	NUDT15
POLR2J	REC8	SETX	SPATA22	TDP2	UBR5	YEATS4	POLG
POLR2K	RECQL	SF3A3	SPIDR	TERB1	UCHL5	YY1	
POLR3C	RECQL4	SF3B3	SPIRE1	TERB2	UFL1	ZBTB1	
POLR3GL	RECQL5	SF3B5	SPIRE2	TERF2IP	UHRF1	ZBTB7A	
POM121	REV1	SFPQ	SPO11	TEX12	UIMC1	ZCWPW1	
POT1	REV3L	SFR1	SPRTN	TEX15	UMPS	ZFYVE26	
PPP4C	REXO4	SGF29	SRSF6	TEX264	UNG	ZMPSTE24	
PPP4R2	RFC1	SHLD1	SSRP1	TFPT	UPF1	ZMYND8	
PPP4R3B	RFC2	SHLD2	STN1	TICRR	UPF3B	ZNF365	
PRDM9	RFC3	SHLD3	STUB1	TIGAR	USP1	ZNF707	
PRIM1	RFC4	SHPRH	STX3	TIMELESS	USP10	ZRANB3	
PRIMPOL	RFC5	SIRT1	SUPT16H	TK2	USP11	ZSWIM7	
PRKCG	RFWD3	SIRT6	SUPT20H	TMED2	USP22	ZWINT	
PRKDC	RHNO1	SIRT7	SUPT3H	TMEM161A	USP28	BRP44	
PRMT6	RIF1	SLF1	SUPT4H1	TNKS1BP1	USP3	COBRA1	
PRPF19	RMI1	SLF2	SUPT5H	TNP1	USP45	DFNA5	
PSMD14	RMI2	SLX1A	SUPT7L	TONSL	USP47	EIF2C4	
PSME4	RNASEH2A	SLX1B	SURF1	TOP3A	USP51	NT5C3	
PTTG1	RNASEH2B	SLX4	SUV39H1	TOPBP1	USP7	RDBP	
PWWP3A	RNASEH2C	SMAD5	SWI5	TP53	UVRAG	TCEB3	
RAD1	RNF111	SMARCA2	SWSAP1	TP53BP1	UVSSA	TH1L	
RAD17	RNF138	SMARCA4	SYCP1	TRAIP	VCP	THOC4	
RAD18	RNF168	SMARCA5	SYCP3	TREX1	VCPIP1	ZNRD1	
RAD21	RNF169	SMARCAD1	TADA1	TREX2	VPS28	MDC 1.00	
RAD21L1	RNF8	SMARCAL1	TADA2B	TRIM28	VPS37B	STN 1.00	
RAD23A	RNMT	SMARCB1	TADA3	TRIP12	VPS37D	KHDC3L	
RAD23B	RPA1	SMARCC1	TAF10	TRIP13	VPS72	MRE11A	
RAD50	RPA2	SMARCC2	TAF12	TRPC2	WAS	HFM1	
RAD51	RPA3	SMARCD1	TAF13	TRRAP	WDHD1	CCNH	
RAD51AP1	RPAIN	SMARCD2	TAF1C	TSG101	WDR48	NUDT1	
RAD51B	RPS3	SMARCD3	TAF2	TTC5	WRAP53	RNF4	
RAD51C	RRM1	SMARCE1	TAF4	TTF2	WRN	FANCE	
RAD51D	RRM2B	SMC1A	TAF5	TWIST1	WRNIP1	DNPH1	
RAD52	RTEL1	SMC2	TAF5L	TYMS	XAB2	GTF2E2	

Supplementary Table 2: List of Unfolded protein response (UPR) genes

Abca7	Cdk5rap3	Dnajb9	Erp27	Ifng	Pik3r1	Syvn1	Vapb
Abcb10	Chac1	Dnajc3	Erp44	Igtp	Pik3r1	Tbl2	Wfs1
Agr2	Comp	Dnajc10	Faf2	Jkamp	Pmp22	Thbs1	Xbp1
Amfr	Cops5	Edem1	Fbxo6	Manf	Ppp1r15a	Thbs4	Yod1
Atf3	Creb3	Edem2	Ficd	Mfn2	Ptpn1	Tm7sf3	
Atf4	Creb3l1	Edem3	Herpud1	Nck1	Ptpn2	Tmbim6	
Atf6	Creb3l2	Eif2a	Herpud2	Nck2	Qrich1	Tmed2	
Atf6b	Creb3l3	Eif2ak2	Hsf1	Nfe2l2	Rhbdd1	Tmem33	
Bag3	Creb3l4	Eif2ak3	Hsp90aa1	Optn	Rpap2	Tmem129	
Bak1	Dab2ip	Eif2ak4	Hspa1a	Os9	Rpap2	Tmtc4	
Bax	Daxx	Eif2s1	Hspa4l	Pacrg	Selenos	Tram1	
Bfar	Ddit3	Erlec1	Hspa5	Parp6	Serp1	Ube2j2	
Bhlha15	Ddrgk1	Ermp1	Hspb1	Parp8	Serp2	Ubxn4	
Bok	Derl1	Ern1	Hspb8	Parp16	Stc2	Ufl1	
Casp12	Derl2	Ern2	Hsph1	Pdia6	Stt3b	Umod	
Ccnd1	Derl3	Ero1a	Hyou1	Pigbos1	Stub1	Upf2	

Supplementary Table 3: List of integrated stress response (ISR) genes

Abca7	Dele1	Eif2s1	Map3k20	Oma1	Rpap2
Agr2	Eif2ak1	Gcn1	Nck1	Ptpn1	Tmed2
Atf4	Eif2ak3	Igtp	Nck2	Ptpn2	Tmem33
Bok	Eif2ak4	Impact	Nfe2l2	Qrich1	

Supplementary Table 4: List of Amino acid transport (AAT) genes

Abat	Gja1	Myo6	Slc1a3	Slc7a3	Slc25a15	Syt4
Abcc8	Gnat2	Nat3	Slc1a4	Slc7a4	Slc25a18	Tnf
Ace2	Grik1	Nf1	Slc1a5	Slc7a5	Slc25a22	Trh
Adora1	Grin2b	Nfe2l1	Slc1a6	Slc7a6	Slc25a26	Trpc4
Adora2a	Grm1	Nfkbie	Slc1a7	Slc7a7	Slc25a29	Trpv1
Agt	Grm2	Nherf1	Slc3a1	Slc7a8	Slc25a38	Tspo2
Apba1	Grm7	Npy5r	Slc3a2	Slc7a9	Slc25a44	Ttyh1
Arg1	Hrh3	Nr3c1	Slc6a1	Slc7a10	Slc32a1	Ttyh2
Arg2	Htr1a	Ntrk2	Slc6a2	Slc7a11	Slc36a1	Ttyh3
Arhgef11	Htr1b	Ntsr1	Slc6a3	Slc7a12	Slc36a2	Ucp2
Arl6ip1	Htr2c	P2rx7	Slc6a4	Slc7a13	Slc36a3	Vps54
Arl6ip5	Htr6	Pak1	Slc6a5	Slc7a14	Slc36a4	Xk
AU018091	Il1b	Pdpn	Slc6a6	Slc7a15	Slc38a1	
Avp	Il1rn	Per2	Slc6a7	Slc11a1	Slc38a2	
Avpr1a	Itgb1	Pianp	Slc6a8	Slc12a2	Slc38a3	
Bdnf	Kcnj8	Prkcd	Slc6a9	Slc13a3	Slc38a4	
Best1	Kcnj10	Prkg1	Slc6a11	Slc15a1	Slc38a5	
Cacna1a	Kcnk1	Psen1	Slc6a12	Slc15a4	Slc38a6	
Cacnb4	Kcnk2	Rab3gap1	Slc6a13	Slc16a2	Slc38a7	
Cck	Kmo	Rgs2	Slc6a14	Slc16a10	Slc38a8	
Cln3	Lep	Rgs4	Slc6a15	Slc17a5	Slc38a9	
Cln8	Llgl2	Septin2	Slc6a16	Slc17a6	Slc38a10	
Cltrn	Lrp5	Sfxn1	Slc6a17	Slc17a7	Slc38a11	
Ctns	Lrrc8a	Sfxn2	Slc6a18	Slc17a8	Slc43a1	
Dpysl2	Lrrc8b	Sfxn3	Slc6a19	Slc22a2	Slc43a2	
Dtnbp1	Lrrc8c	Sfxn3	Slc6a20a	Slc22a4	Slc47a1	
Epm2a	Lrrc8d	Sfxn4	Slc6a20b	Slc22a15	Slc66a1	
Gabbr1	Lrrc8e	Sfxn5	Slc6a21	Slc25a2	Snca	
Gfap	Mfsd12	Slc1a1	Slc7a1	Slc25a12	Stxbp1	
Gipc1	Myc	Slc1a2	Slc7a2	Slc25a13	Sv2a	

Supplementary Table 5: List of Peroxidase activity (PACT) genes

Alox5ap	Gpx6	Gstp2	Hbb-bh0	Mb	Prdx5	Tpo
Cat	Gpx7	Gstp3	Hbb-bh1	Mgst1	Prdx6	Txndc17
Cp	Gpx8	Gstp-ps	Hbb-bh2	Mgst2	Prdx6b	Txnrd1
Cygb	Gsta1	Gstt1	Hbb-bs	Mgst3	Prxl2b	Upk3bl
Epx	Gsta2	Gstt2	Hbb-bt	Mpo	Ptges	
Gpx1	Gsta5	Hba-a1	Hbb-y	Park7	Ptgs1	
Gpx2	Gsta13	Hba-a2	Hbq1a	Prdx1	Ptgs2	
Gpx3	Gstk1	Hba-x	Hbq1b	Prdx2	Pxdn	
Gpx4	Gstm7	Hbb-b1	Lpo	Prdx3	Selenof	
Gpx5	Gstp1	Hbb-b2	Ltc4s	Prdx4	Sesn2	

Supplementary Table 6: List of Nrf2 target genes

ABCB6	UGT1A1	SRXN1	PGD	BLVRA	PSMA1	BCL2
ABCC1	GCLC	TXN1	TALDO1	BLVRB	PSMB5	
ADH7	GCLM	G6PD	TKT	FTH1	SQSTM1	
CBR1	GPX1	HDK1	ACOT7	FTL1	ATG5	
CYP1B1	GSR1	IDH1	ACOX1	HMOX1	ATG7	
EPHX1	PRDX1	ME1	SCD2	ATF4	LC3B	

Supplementary Table 7: List of Eukaryotic translation factor (ETF) genes

EIF1	EIF3C	EIF3M	EIF5	CPSF6	PCF11	POLR3D
EIF1A	EIF3D	EIF4A1	EIF5B	CPSF7	SCAF4	POLR3E
EIF2A	EIF3E	EIF4A2	EFTu	PABPN1	SCAF8	POLR3F
EIF2B1	EIF3F	EIF4B	EFTS	PABPC1	RPRD1A	POLR3G
EIF2B2	EIF3G	EIF4E	EFGM	PAPOLA	RPRD1B	POLR3H
EIF2B3	EIF3H	EIF4F	EEFSEC	PAPOLG	RPRD2	MRRF
EIF2B4	EIF3I	EIF4G1	CPSF1	PAPOLB	CAVIN1	ABCE1
EIF2B5	EIF3J	EIF4G2	CPSF2	XRN2	POLR3A	MCTS1
EIF3A	EIF3K	EIF4G3	CPSF3	TTF2	POLR3B	DENR
EIF3B	EIF3L	EIF4H	CPSF4	SETX	POLR3C	

Supplementary Table 8: Used Oligonucleotides

Mouse Bcl6 CHIP qPCR Forward:	TGTTTGCTTGGGGAGATGATG
Mouse Bcl6CHIP qPCR Reverse:	CTGACAATGCCAGCAAACCAT
Mouse Cirbp CHIP qPCR Forward:	GAGGGGCAAGCAAGATGACT
Mouse Cirbp CHIP qPCR Reverse:	ATTTAACTGAGGCGCATTTGTG
Mouse Uba52 CHIP qPCR Forward:	ATTCTCGCCAGGACCCAAC
Mouse Uba52 CHIP qPCR Reverse:	AGAGAAAGAGGACAGCGGAAC
Mouse Ebf1 CHIP qPCR Forward:	TGGCTGTGATGGAAGTTGGTT
Mouse Ebf1 CHIP qPCR Reverse:	CCTTCTCCTGCTCCGCAAA
Mouse Ebf1 firefly vector clone Forward:	CCGCCGCTCGAGGGTCAATAGGCCTGAGATGGG
Mouse Ebf1 firefly vector clone Reverse:	CCCCCCAAGCTT TTCACTCGGTCACTACTCTCCA
Mouse cirbp firefly vector clone Forward:	CCGCCGCTCGAG ACTGAGTTGGGTGCAGGAAA
Mouse cirbp firefly vector clone Reverse:	CCCCCCAAGCTT TGCTACCTCAATGAGACTCCC
Mouse uba52 firefly vector clone Forward:	CCGCCGCTCGAG GCTAGCCTAGTACTCACCGC
Mouse uba52 firefly vector clone Reverse:	CCCCCCAAGCTT CATGCACGACGCCGAAG
Mouse Cux2 ish plasmid cloning forward:	GGTCCTCACCGACAACAACC
Mouse Cux2 ish plasmid cloning reverse:	TGGACTTGAAACTCAGAGGCT
Mouse Atf4 qPCR forward:	TCGGCCCAAACCTTATGACC
Mouse Atf4 qPCR reverse:	TGGCTGCTGTCTTGTTTTGC
Mouse Cacna1i qPCR forward:	GAGGATGCCCACCAGTTCAC
Mouse Cacna1i qPCR reverse:	GATGGAGCCAGACAGGTTGT
Mouse Cirbp qPCR forward:	TCTCCGAAGTGGTGGTGGTA
Mouse Cirbp qPCR reverse:	CATCATGGCGTCCTTAGCGT
Mouse Uba52 qPCR forward:	CAGAGGCTGATATTCGCGGG
Mouse Uba52 qPCR reverse:	CTTCTGGGCAAGCTGACGAA
Mouse Ebf1 qPCR forward:	GTGGAGATTGAGAGGACGGC
Mouse Ebf1 qPCR reverse:	TCCTGTTCTGTCCGTATCCC
Mouse Ano3 qPCR forward:	TCCTTTGCTGACCTCAGTGAC
Mouse Ano3 qPCR reverse:	GCTTTGTCTTGGATGCAGGC
Mouse Eda2r qPCR forward:	TTGTTGCACTGGTGGGAAGT
Mouse Eda2r qPCR reverse:	CCAGGTGGTACGGGGAAAAG
Mouse ccng1 qPCR forward:	CTTCTGTGCTGGCGCTATCT
Mouse ccng1 qPCR reverse:	AGGTCAAATCTCGGCCACTT
Mouse cdkn1a qPCR forward:	GCAAAGTGTGCCGTTGTCTC
Mouse cdkn1a qPCR reverse:	CGTCTCCGTGACGAAGTCAA
Mouse Zmat3 qPCR forward:	TGGCCACCAGGAGGAATATG
Mouse Zmat3 qPCR reverse:	AACTGGCCACTTGGAGTCAC
Mouse Dglucy qPCR forward:	GGTTCCAGAAGTCCATGCCA
Mouse Dglucy qPCR reverse:	GGGTCTACGGCAATTGTGGA
Mouse Actb qPCR forward:	GCAAGTGCTTCTAGGCGGAC
Mouse Actb qPCR reverse:	AAGAAAGGGTGTAAAACGCAGC
Mouse Setx qPCR forward:	CCTGTTCCGTAAACCTGGCT
Mouse Setx qPCR reverse:	GAACCCATGGAAGAGGGTGG

Supplementary Table 8 (continued): Used Oligonucleotides

Mouse Atf3 qPCR forward:	CCTCAGAAGTCAGTGCGACC
Mouse Atf3 qPCR reverse:	GAGGACATCCGATGGCAGAG
Mouse Atf5 qPCR forward:	CTTGTCAACCCTGCCTGTCC
Mouse Atf5 qPCR reverse:	CTTCTTTTGCTTGCGGTCCC
Mouse Atf6 qPCR forward:	GGAGTCGACGTTGTTTGCTG
Mouse Atf6 qPCR reverse:	GGTCTGACTCCCAAGGCATC
Mouse Ddit3 qPCR forward:	CAGGAGAACGAGCGGAAAGT
Mouse Ddit3 qPCR reverse:	TGACCATGCGGTCGATCAG
Mouse Ppp1r15a qPCR forward:	CTCTGCCTTGTGGAAGCTGA
Mouse Ppp1r15a qPCR reverse:	CAGCTGTGCGTTCCATTTCC
Mouse Xbp1 qPCR forward:	GCAGCAAGTGGTGGATTTGG
Mouse Xbp1 qPCR reverse:	CAGGATCCAGCGTGTCCATT
Mouse P4hb qPCR forward:	GAGGACAACGTCCTGGTGTT
Mouse P4hb qPCR reverse:	CTTCAGTTTTGCGGCAGCTT
Mouse Slc1a3 qPCR forward:	CCTCTTCTTGATCGCAGGGA
Mouse Slc1a3 qPCR reverse:	CAGGGGTTCTTCCGGGTTAC
Mouse Slc1a4 qPCR forward:	CAGCGCTGTGCTAGCTTTT
Mouse Slc1a4 qPCR reverse:	AGGTAAGTGATCTGGGTGCG
Mouse Slc7a1 qPCR forward:	CCCTTTGTCCCCGTACTTCC
Mouse Slc7a1 qPCR reverse:	GCTTCCTCACTGTGCCAGAT
Mouse Slc38a2 qPCR forward:	GGCATTCAATAGCACCGCAG
Mouse Slc38a2 qPCR reverse:	ACGTTCATCATCCGTCTCCG
Mouse Cirbp shRNA1	CGTCC TTCC ATGG CTGT AATT
Mouse Cirbp shRNA2	CAGAG ACAG CTAT GACA GTTA
Mouse Ebf1 shRNA1	CCCTG AAAT GTGC CGAG TATT
Mouse Ebf1 shRNA2	GCGCG ACTG TGAT CATC ATAG
Mouse Uba52 shRNA1	CCAGA AGTA CAAC TGTG ACAA
Mouse Uba52 shRNA2	CGAGA ATGT CAAG GCCA AGAT
Mouse Slc1a3 shRNA	CCATGTGCTTCGGTTTCGTGA
Mouse Slc1a4 shRNA	GCTCAGTTTGAGTAAACCATA
Mouse Slc7a1 shRNA	CCTCACAATCTCTCCACTCAT
Mouse Slc38a2 shRNA	ATGACGAGCAAATTGGTGAAG
